# Dermatoglyphics in kidney diseases: a review

**DOI:** 10.1186/s40064-016-1783-7

**Published:** 2016-03-08

**Authors:** Buddhika T. B. Wijerathne, Robert J. Meier, Sujatha S. Salgado, Suneth B. Agampodi

**Affiliations:** Department of Forensic Medicine, Faculty of Medicine and Allied Sciences, Rajarata University of Sri Lanka, Saliyapura, 50008 Sri Lanka; Department of Anthropology, Indiana University, Bloomington, IN USA; Department of Anatomy, Faculty of Medicine, University of Kelaniya, Ragama, Sri Lanka; Department of Community Medicine, Faculty of Medicine and Allied Sciences, Rajarata University of Sri Lanka, Saliyapura, Sri Lanka

**Keywords:** Kidney disease, Dermatoglyphics, Wilms tumour, Review

## Abstract

Kidney diseases are becoming a major cause of global burden with high mortality and morbidity. The origins of most kidney diseases are known, but for some the exact aetiology is not yet understood. Dermatoglyphics is the scientific study of epidermal ridge patterns and it has been used as a non-invasive diagnostic tool to detect or predict different medical conditions that have foetal origin. However, there have been a limited number of studies that have evaluated a dermatoglyphic relationship in different kidney diseases. The aim of this review was to systematically identify, review and appraise available literature that evaluated an association of different dermatoglyphic variables with kidney diseases. This review is reported according to the Preferred Reporting Items for Systematic Reviews and Meta-Analyses checklist. The PubMed^®^ (Medline), POPLINE, Cochrane Library and Trip Database and grey literature sources such as OpenGrey, Google Scholar, and Google were searched to earliest date to 17 April 2014. Of the 36 relevant publications, 15 were included in the review. Of these studies, there are five case reports, seven case series and three comparative studies. Possible association of dermatoglyphics with Wilms tumor (WT) had been evaluated in two comparative studies and one case series that found fewer whorls and a lower mean total ridge count (TRC). Another study evaluated adult polycystic kidney disease (APCD) type III that revealed lower TRC means in all cases. All other case series and case reports describe dermatoglyphics in various kidney disease such as acro-renal-ocular syndrome, potter syndrome, kabuki makeup syndrome, neurofaciodigitorenal syndrome, syndactyly type V, ring chromosome 13 syndrome, trisomy 13 syndrome and sirenomelia. It is evident that whorl pattern frequency and TRC have been used widely to investigate the uncertainty related to the origin of several kidney diseases such as WT and APCD type III. However, small sample sizes, possibly methodological issues, and discrepancy in the make up between cases and control groups limits interpretation of any significant findings. Future studies with proper protocol, adequate cases, and control groups may provide stronger evidence to resolve uncertainty related to the aetiology of kidney diseases.

## Background

Kidney diseases are becoming a global burden (The Lancet 2013) with between 8 and 16 % of the world’s population suffering from chronic kidney disease (CKD) (Jha et al. 2013). Further, there is an increased concern of acute kidney injury as well (Lameire et al. 2013). Kidney diseases categorized as hereditary (e.g. polycystic kidney disease, Alport syndrome, etc.), congenital (malformation of urinary tract casing disease), and acquired kidney diseases (more common) (HealthCentral). There are several identifiable causes of kidney diseases, including diabetes, hypertension, glomerulonephritis and genetically inherited diseases (Colledge et al. 2010). However, in several countries, exact aetiology of some CKD patients is unknown (Jha et al. 2013).

Dermatoglyphics is the study of the epidermal ridge patterns on the skin of the fingers, palms, toes, and soles (Cummins and Midlo 1961). Epidermal patterns start to develop during the sixth and seventh weeks of intrauterine life, and are fully formed by the end of the second trimester (Blackwell 1994). These anatomical structures have been used widely in the field of anthropology (Meier 1980) in addition to also being used in medicine and genetics as a valuable diagnostic tool (Holt 1973; Reed and Opitz 1981; Shiono 1986). There is a popularity of using dermatoglyphics as a non-invasive diagnostic tool to detect and predict different medical conditions that occur in early life (Kumar and Manou 2003; Fuller 1973; Cvjeticanin et al. 2009; Pakhale et al. 2012; Gupta and Karjodkar 2013), especially in clinical settings with minimal high tech diagnostic capabilities. These studies were based on the hypothesis “if growth of the limbs is disturbed in very early fetal life changes in the epidermal ridge configurations are likely” (Schaumann and Johnson 1982; Babler 1991; Blackwell 1994). Therefore, dermatoglyphic association of various diseases with ectodermal origin have been extensively evaluated.

In addition, the relationship between different dermatoglyphic traits and the diseases of the bodily structures that originate primarily from mesoderm have been widely evaluated. The dermatoglyphics of diseases such as red cells (thalassemia, sickle cell anaemia), lymphocytes (acute lymphocytic leukaemia), cardiac muscles and vessels (ischemic heart disease, hypertension, rheumatic heart disease, and dilated cardiomyopathy) are evaluated in the literature (Annapurna et al. 1978; Sanyal 1978; Edelstein et al. 1991; Polzik and Sidorovich 1991; Palyzová et al. 1991; Oladipo et al. 2007; Dogramaci et al. 2009; Solhi et al. 2010; Bukelo et al. 2011; Ramesh et al. 2012; Fayrouz et al. 2012; Wijerathne et al. 2015).

The kidney is an anatomical structure that primarily originates from the mesoderm (Gilbert 2000; Murer et al. 2007). There are a limited number of studies that have evaluated a dermatoglyphic relationship in different kidney diseases (Curró et al. 1982; Hauser et al. 1984; Abd Allah et al. 2011). Therefore, as a start, we conducted this review to identify and appraise the different dermatoglyphic variables that might be associated with kidney diseases. These findings are an important undertaking at this time and serve as the basis for conducting this line of research.

## Methods

This review is reported according to the Preferred Reporting Items for Systematic Reviews and Meta-Analyses (PRISMA) checklist (Moher et al. 2009).

### Search strategy

We searched the following electronic databases earliest inclusive dates to April 17, 2014. The databases included PubMed^®^ (Medline), POPLINE, Cochrane Library and Trip Database. In addition, we searched the grey literature sources; namely, OpenGrey, Google Scholar, and Google. We did not restrict the searches based on language, year of publication, or publication status.

A Boolean search strategy was constructed in Medline database using the following MeSH (medical subject headings) terms:

Dermatoglyphics [MeSH Terms] AND (“Kidney Diseases” [MeSH Terms] OR “Kidney Neoplasms” [MeSH Terms] OR “Kidney” [MeSH Terms] OR “Kidney/abnormalities” [MeSH Terms] OR “Kidney/embryology” [MeSH Terms] OR “Kidney Failure, Chronic” [MeSH Terms] OR “Renal Insufficiency, Chronic” [MeSH Terms] OR “Acute Kidney Injury” [MeSH Terms] OR “Kidney/growth and development” [MeSH Terms]).

A search of other databases and grey literature was conducted with the same MeSH terms.

### Eligibility criteria and study selection

Available full text articles were obtained for all studies. For articles where full texts were not available, the abstract and title were evaluated. Initially the full texts or title and abstracts were screened by BTBW based on the following inclusion and exclusion criteria.

### Inclusion criteria

- Peer reviewed journal articles that describe dermatoglyphic traits in different kidney disease;
- Studies conducted on human subjects.

### Exclusion criteria

- Review articles;
- Editorials.

Later all potential studies were independently reviewed by SBA and SSS for accuracy. Disagreements were discussed with a third reviewer RJM for final selection of studies to be included in the review.

### Data extraction

From every included study the following details were extracted, age, sex, region, ethnicity, type of kidney disease, type of kidney abnormality and dermatoglyphic characteristics. At first, data from case reports, case series and case control studies were extracted and placed into three separate tables by BTBW. Then, these tables were checked by two other authors (SBA, SSS) for accuracy. A third author RJM finally reviewed selected studies to ensure uniformity.

## Results

**Fig. 1:**
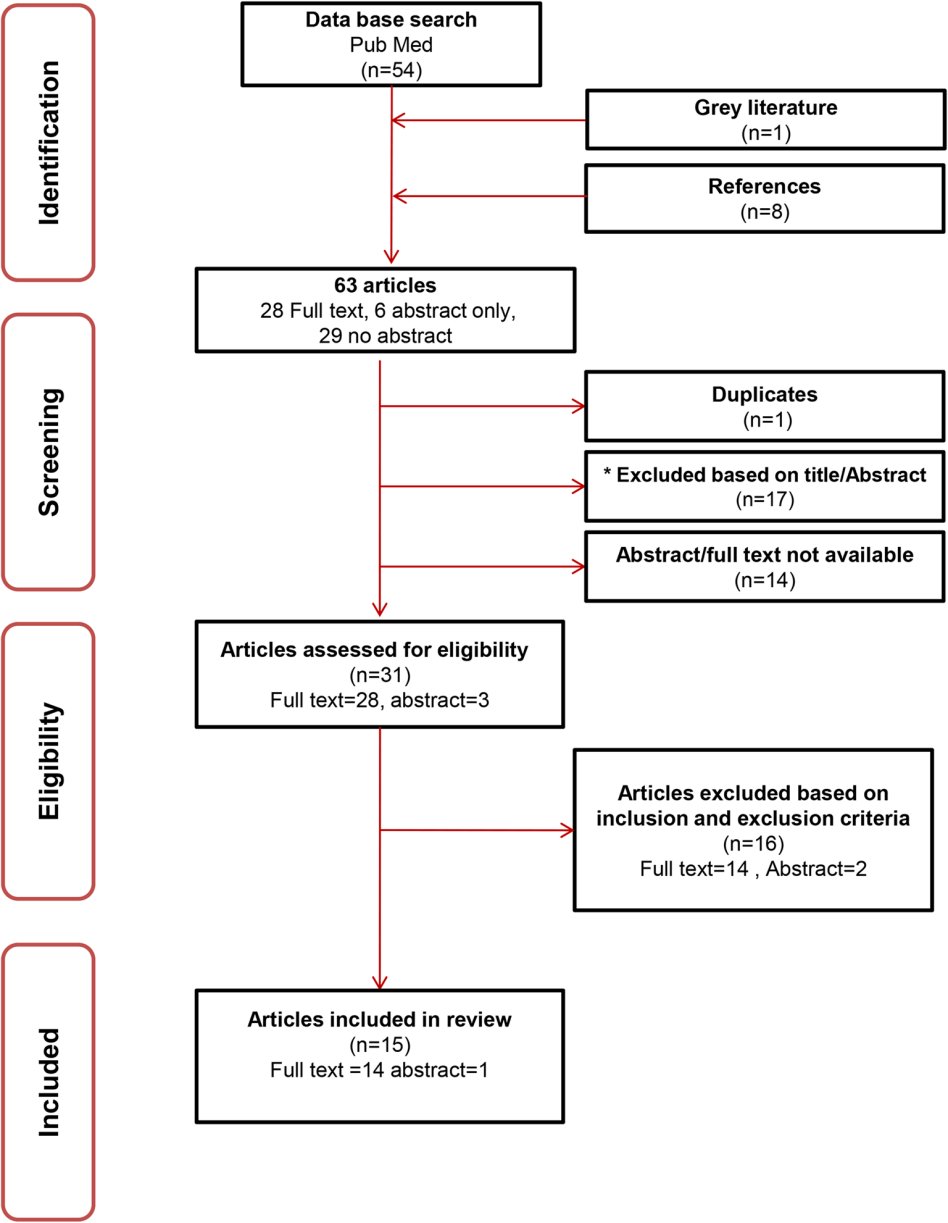
Summary of evidence search and selection. Asterisk represents Online Mendelian Inheritance in Man^®^ (http://omim.org) and Orphanet (http://www.orpha.net) database referred for disease characteristics

The literature search identified 63 articles (Fig. 1). Of these, a systematic search of PubMed databases yielded 54 studies. One study was identified through the grey literature search. Eight studies were found through the manual searches of the reference lists of the retrieved full text articles.

Out of these 63 articles, 14 publications were excluded based on title or abstract (Online Mendelian Inheritance in Man^®^ (http://omim.org) and Orphanet (http://www.orpha.net) database referred for disease characteristics), one was a duplicate and an abstract or full text was unavailable for 14 titles.

Finally, 31 articles (28 full texts, three abstracts) were assessed which led to the inclusion of 15 studies [one study is an abstract only (Hauser et al. 1984)].

Of these studies, there are five case reports, seven case series and three comparative studies.

The extracted data are shown in Tables 1, 2 and 3, respectively.

**Table 1:** Case series

Author	Case no.	Gender	Age (years)		Ethnicity	Country	Disease	Kidney anomalies	Dermatoglyphic variables
			Case	Parents					
Fraumeni et al. (1967)	1	F	11½	NR	NR	USA	Wilms tumor (+congenital hemiarthropathy)	Nephroblastoma Histologically confirmed	Dermatoglyphic variables of palms and fingers were within normal limits and found no significant differences between the two sides
	2	F	16	NR	NR				
	4	F	2	NR	NR				
	5	F	7½	NR	NR				
Juberg et al. (1975)	1	M	2	NR	NR	USA	Wilms tumor	Histologically confirmed nephroblastoma	No dermatoglyphic abnormalities. Dermatoglyphics used to confirm monozygotic twining
Freire-Maia et al. (1982)	1	M	7 4/12 First child	M = 38 F = 25	Brazilians (Caucasian ancestry)	Brazil	Neurofaciodigitorenal (NFDR) syndrome	Both kidneys with normal execratory function	Digital dermatoglyphics Qualitative Right hand: UL in digit (1, 2, 4, 5) and RL in digit 3 Left hand: DL in digit (1), UL in digit (2, 5), A in (3 and 4) Palmar dermatoglyphics Qualitative Both hands: axial triradius (t), no thenar pattern, no hypotherner, no interdigital, distal triradii (c absent) Quantitative Right hand: atd angle = 44°, ulnarity index = 0.57 Left hand: atd angle = 41°, ulnarity index = 0.48
	2	M	5 Second child	M = 40 F = 27				Absence of L kidney, anteversion of R kidney	Digital dermatoglyphics Qualitative Right hand: UL in digit (4, 5), RL in 1 and A in (2, 3) Left hand: UL in digit (3, 4, 5), RL in 2, W in 1 Palmar dermatoglyphics Qualitative Right hand: no interdigitalpattern, distaltriradii (c absent) Left hand: interdigital (Ld in ID 4) Both hands: axial triradius (t), no thenar pattern, no hypotherner pattern Quantitative Right hand: atd angle = 32°, ulnarity index = 0.67 Left hand: atd angle = NR, ulnarity index = NR
Passarge (1965)	1	M	34 weeks	M = 26	NR	NR	Potter’s syndrome	Kidneys and ureters were absent and only a rudimentary bladder was found	Palmar dermatoglyphics Qualitative Right hand: single palmar crease Left hand: single palmar crease
	2	M	32 week 2 h old	M = 24	NR			Absent kidney Ureters and bladder were normal	NR
	3	F	36 week	M = 26	NR			The kidneys were large, and were cystic and dysplastic. The ureters and the bladder were norma	Palmar dermatoglyphics Qualitative Right hand: single transverse palmar Creases Left hand: single transverse palmar creases
Robinow et al. (1982)	4	F	9	M = 30	Puerto Rican	USA	Syndactyly type V	Hypoplastic pelvic kidney on the left Both collecting systems were dysmorphic	Digital dermatoglyphics Qualitative Right hand: A in digit 1–3, UL in digit 4–5 Left hand: A in all digits Both hands: lacked distal Single transverse creases, distally displaced axial triradii and greatly reduced number of distal palmar triradii
Halal et al. (1984)	1	M	45	NR	NR	Canada	Acro–renal–ocular syndrome	Left crossed renal ectopia without fusion. Urinary tract anomaly	Digital dermatoglyphics Qualitative Right hand: W in all digit Left hand: W in (1–4), UL in 5 Quantitative Both hands: TRC = 210 (high) Palmar dermatoglyphics Qualitative Right hand: Palmar triradius t^1^, MLF: 7–11.9.7.4-t′-0.0.0.L^d^.L^d^ Left hand: Palmar triradius t^1^, thenar/I1 pattern, MLF: 7–9.9.5″.3-t′-.W/0.0.L^d^.L^d^
	2	M	25	NR	NR			Left paraurethral diverticulum	Digital dermatoglyphics Qualitative Right hand: UL in (3–5), A in 2nd, W in 1st Left hand: UL in all digits Quantitative Both hands: TRC = 131 Palmar dermatoglyphics Qualitative Right hand: Palmar triradius t^1^, MLF: 9.7.5″.3-t-0.0.0.0.L^d^ Left hand: Palmar triradius t^1^, Ulnar pattern ID area IV, MLF: 9.7.5″.3-t-0.0.0.0.L^d^
	3	F	24	NR	NR			Scaral right renal ectopia	Digital dermatoglyphics Qualitative Right hand: W in (1, 2, 4, 5), UL in 3rd Left hand: UL in (1–3), W in (4, 5) Quantitative Both hands: TRC = 199 (high) Palmar dermatoglyphics Qualitative Right hand: Palmar triradius t^1^, MLF: 7.5″.5″.3-t′-0.V.0.0.L^d^ Left hand: Palmar triradius t^1^, MLF: 5″-9.9.5″.3-t′-0.0.0.L.^d^.L^d^ Both hands: thenar exist of A line
	4	F	22	NR	NR			Malrotated right kidney	Digital dermatoglyphics Qualitative Right hand: UL in 2–5), absent D1 Left hand: W in (1, 2), UL in (3–5) Quantitative Both hands: TRC = 172 Palmar dermatoglyphics Qualitative Right hand: Palmar triradius t1, absence of axial triradius MLF: 9.X.5″.4-abs-0.0.0.0.0 Left hand: Palmar triradius t^1^ MLF: 9–9.7.5″, I-t′-0.0.0.0.L^d^.L^d^ Both hands: thenar exist of A line
	5	F	21	NR	NR			Left kidney (10 cm)is slightly smaller than right kidney (12.5) and malrotated	Digital dermatoglyphics Qualitative Right hand: UL in (1–3), W in (4, 5) Left hand: LH: W in (2, 4, 5), UL in 4, A in 1 Quantitative Both hands: TRC = 166 Palmar dermatoglyphics Qualitative Right hand: Palmar triradius t^1^ MLI: 9.7.5″.3-t′-0.0.0. L^d^.0 Left hand: Palmar triradius t1 MLI: 7.9.5″.3-t-0.0.0.L^d^.L^d^
	6	F	17	NR	NR			Left crossed renal ectopia without fusion urinary tract anomaly, VUR in the ectopic kidney	Digital dermatoglyphics Qualitative Right hand: UL in (2–5), W in 1 Left hand: UL in all digit Quantitative Both hands: TRC = 144 Palmar dermatoglyphics Qualitative Right hand: Palmar triradius t1 MLF: 7.5″.5′. I-t′-V.0.0.0.L^d^ Left hand: Palmar triradius t1 Central pocket whorl pattern in ID area IV MLF: 7.5″.5″. 3-t′-0.0.0.0.W^cpd^ Both hands: thenar exist of A line
	7	F	2 month	NR	NR			VUR grade 11A	Digital dermatoglyphics Qualitative Right hand: UL in (3, 5), W in (1, 4), A in 2 RL pattern in extra thumb Left hand: UL in (3, 4), W in (1, 5), RL in 2 Palmar dermatoglyphics Qualitative Right hand: Palmar triradius t1 MLF: 7-9.abs.5″. I-t′-L^r^.0.0.0.L^d^ Left hand: Palmar triradius t′ MLF: 7-9.abs.5″. I-t′-L^r^.0.0.0.L^d^ Both hands: Thenar exist of A line, absent C
Philip et al. (1992)	1	M	12	M = 22 F = 32	French	Japan	Kabuki make-up (Niikawa–Kuroki) syndrome	Horseshoe kidney	Digital dermatoglyphics Qualitative Both hands: fingertip ulnar loop10/10 Palmar dermatoglyphics Qualitative Right hand: missing triradius c, hypothenar loop Left hand: missing triradius c, hypothenar loop
	2	M	8½	M = 23 F = 29	German			Abnormality +	Digital dermatoglyphics Qualitative Both hands: fingertip ulnar loop 9/10 Palmar dermatoglyphics Qualitative Right hand: missing triradius d, hypothenar loop Left hand: missing triradius d, hypothenar loop
	3	M	3½	M = 32 F = 38	French			Mild urinary reflux	Digital dermatoglyphics Qualitative Both hands: fingertip ulnar loop9/10 Palmar dermatoglyphics Qualitative Right hand: hypothenar loop, interdigital triradius bc or cd Left hand: hypothenar loop, missing triradius c

*NR* not reported, *A* arch, *W* whorl, *UL* ulnar loop, *RL* radial loop, *DL* double loop, *TRC* total finger ridge count, *MLF* main line formula, *M* male, *F* Female, *VUR* vesicoureteral reflux, *ID* interdigital

**Table 2:** Case reports

Author	Gender	Age (years)		Ethnicity	Country	Disease	Kidney anomalies	Dermatoglyphic variables
		Case	Parents					
Hoo et al. (1974)	M	14 month	M = 30 F = 31	NR	Germany	The ring chromosome 13	Agenesis of right kidney	Digital dermatoglyphics Qualitative Right hand: query loop pattern in digit 1, UL in digit 2–5 Left hand: query whorl pattern in digit 1, UL in digit 2–3, DL in digit 4, W in digit 4 Quantitative Both hands: TRC 113 Palmar dermatoglyphics Qualitative Right hand: 11.9.7.5′-ttu-L^r^.0.0.L.0 Left hand: 11.X.7.3-ttu-L^r^.0.0.0.0 Plantar dermatoglyphics Qualitative Right sole: L^d^.0.Ld.0.0 Left sole: L^d^.0.L^d^.0.0
Pettersen (1979)	M	2	NR	NR	USA	Trisomy 13 syndrome	Horseshoe kidney with slight pyelocalyceal dilation glomerular and tubular cysts	Digital dermatoglyphics Qualitative Right hand: UL in digit 1, 3–4, RL in digit 2 and 5 Left hand: UL in digit 1, 3, 5, RL in digit 2, W in digit 4 Palmar dermatoglyphics Qualitative Right hand: Inter digital areas t, t’’.0.W 0.0.L^d^.0 Left hand: interdigital areas t, t’’.0 W 0.0.V.L^d^.0 Plantar dermatoglyphics Qualitative Right hallucal area: F^S^ Left hallucal area: vF^S^
Crawfurd et al. (1966)	Male karyotype	Stillborn	M = 29	NR	UK	Sirenomelia	The kidneys, ureters, and bladder were apparently absent, a small round pink structure of 0.5 cm. Posterior wall of the pelvis. Well-defined cortex and medulla, many of the glomeruli appeared immature, and the tubules and collecting ducts were poorly formed with a few microcysts	Digital dermatoglyphics Qualitative Right hand: W in all five fingers Left hand: W in digit 1–4, UL in digit 5 Palmar dermatoglyphics Qualitative Right hand: 3rd interdigitalpattern, a single axial triradius in the usual t position, no thenar pattern Left hand: 3rd and a 4th inter digital pattern, a similar single axial triradius to that on the right, no thenar pattern Dermatoglyphics on abnormal limb ridges were poorly formed, running in horizontal circles proximally, longitudinal lines distally with a single central triradius at the junction of the circular and longitudinal ridges
Iwama et al. (1987)	M	4 months	M = 28 F = 26	NR	Japan	Kabuki makeup syndrome	Megaureter and hypo-plastic L-shaped kidneys	Digital dermatoglyphics Qualitative Right hand: UL in all fingers Left hand: UL in digit 2–5, W in digit 1 Palmar dermatoglyphics Qualitative Right hand: hypothenar loop pattern, absence of digital triradius c and d Left hand: hypothenar loop pattern, absence of digital triradius c and d
Jancar (1969)	M	33 (calculated)	NR	NR	UK	Potter’s syndrome	Large right sided hydroneprosis with considerable loss of renal tissue, Congenital stricture of upper ureter with kink at pelvi-ureteric junction, chronic pyelonephritis observed during the study	Digital dermatoglyphics Qualitative Right hand: UL in 3–5, RL in 2, W in 1 Left hand: UL in 1, 4 and 5, RL in 3, TA in 2 Both hands: TRC = 64

*NR* not reported, *A* arch, *W* whorl, *UL* ulnar loop, *RL* radial loop, *DL* double loop, *TRC* total finger ridge count, *MLF* main line formula, *M* male, *F* Female

**Table 3:** Comparative studies

Author	Study group	Number of participant	Gender	Age (years)	Ethnicity	Country	Disease/kidney anomalies	Dermatoglyphic variables
Curró et al. (1982)	Cases	30 unrelated patients	Male = 13	6 month–12 years	NR	Italy	Wilms tumor (histologically confirmed)	Digital dermatoglyphics Qualitative In males, significantly decrease the incidence of whorl (*P* < 0.025) and radial loops (*P* < 0.05), significantly increase the Incidence of arches (*P* < 0.0005) Quantitative Mean PII significantly lower (*P* < 0.02) in WT males; cases = 11 ± 3.78 (mean ± SD) and Control = 14.13 ± 3 (mean ± SD) TRC significantly lower in WT males, cases = 143.53 ± 88.72 (mean ± SD) and controls = 204.22 ± 69.29 (mean ± SD) TRC significantly lower in WT females, cases = 123.93 ± 66.57 (mean ± SD) and controls = 176.29 ± 67.68 (mean ± SD) Palmar dermatoglyphic Maximal atd angle (sum of right and left atd angles); maximal atd angle of female WT patient were higher compared to control (*P* < 0.01) Cummins index; significantly lower in both females (*P* < 0.001) and males (*P* < 0.001) compared to controls
			Female = 17					
	Control	44	Male = 22	NR	NR	Italy		
			Female = 22					
Gutjahr et al. (1975)	Cases	30 WT	Out of all 60 cases	6 months–15 years (average of 5¾ years) for All 60 cases) NR separately for cases	NR	Germany	60 tumor patients (WT = 30, NB = 13, RS = 7, MT = 5, MB = 4 C = 1)	In 30 Wilms tumor patient compared to control Digital dermatoglyphics Qualitative WT: arch = 5.7 %, loop = 59.7 %, whorl = 34.7 %/, normal: arch = 7.9 %, loop = 63.1 %, whorl = 29.0 %) Quantitative Wilms tumor group TRC = 121.9 ± 37.4 compared to 136.4 ± 53.4 Palmar dermatoglyphics Qualitative III interdigital pattern 21.7 % in WT compared to 44 % in general population IV interdigital pattern 36.7 % in WT+ other tumors compared to 60 % expected value Quantitative a–b ridge count 63 % has <78 Plantar dermatoglyphics Qualitative Planter II interdigital patten 31.7 % in WT (both gender) compared to 28 % expected value WT: A = 42.7 %, L = 46 %, W = 11.3 %/, normal: A = 19 %, L = 59.3 %, W = 21.7 % Distally open loops common on great toe
			M = 26					
			F = 34					
			NR separately for cases					
	Control	200 (based on Table 1 in the article)	NR (in the article)	NR	NR	NR		
Hauser et al. (1984)	Cases	9	NRA	NRA	NRA	NRA	adult polycystic kidney disease (APCD) type III	Intrafamilial comparison reviled that their ridge counts on fingers and palms were somewhat lower compared to healthy siblings
	Control	NRA	NRA	NRA	NRA	NRA	NRA	

*NR* not reported, *NRA* not reported in abstract, *A* arch, *W* whorl, *L* loop, *TRC* total finger ridge count, *MLF* main line formula, *M* male, *F* female, *WT* Wilms tumor, *PII* pattern intensity index, *NB* neuroblastoma, *RS* rhabdomyosarcoma, *MT* malignant teratoma, *MB* medulloblastoma, *C* chordoma

### Characteristics of patients with kidney diseases

The patient pool consisted of 28 females, 27 males and one case with male karyotype (Freire-Maia et al. 1982). In addition, there were 30 patients with Wilms tumor (Gutjahr et al. 1975) and nine patients with adult polycystic kidney disease (APCD) type III (Hauser et al. 1984) where sex of the patient was not mentioned. Age ranged from birth at 32 weeks of gestation to 45 years, for all studies that reported this information.

Cases originated from several countries: USA (Fraumeni et al. 1967; Juberg et al. 1975; Pettersen 1979; Robinow et al. 1982), Brazil (Freire-Maia et al. 1982), Canada (Halal et al. 1984), Japan (Iwama et al. 1987; Philip et al. 1992), Germany (Hoo et al. 1974), UK (Crawfurd et al. 1966; Jancar 1969), Italy (Curró et al. 1982), and for two studies the country of origin was not mentioned (Passarge 1965; Hauser et al. 1984).

Ethnicities of the cases are as follow: Brazilians (Caucasian ancestry) (Freire-Maia et al. 1982), French Canadians (Halal et al. 1984), Puerto Rican (Robinow et al. 1982), French (Philip et al. 1992), German (Philip et al. 1992). Ethnicities were not mentioned in several cases (Passarge 1965; Crawfurd et al. 1966; Fraumeni et al. 1967; Jancar 1969; Hoo et al. 1974; Juberg et al. 1975; Pettersen 1979; Curró et al. 1982; Halal et al. 1984; Hauser et al. 1984; Iwama et al. 1987).

### Different kidney diseases and their dermatoglyphic traits

There were several diseases described in these studies that had stated the renal anomalies along with dermatoglyphic examinations.

### Wilms tumur (Fraumeni et al. 1967; Juberg et al. 1975; Curró et al. 1982)

Wilms’ tumor (WT) is the most common renal tumor in childhood and responsible for about 6 % all paediatric cancers (Kalapurakal et al. 2004). The dermatoglyphic variables in WT were described in three studies (two case controls and one case series) and one case series (dermatoglyphics used to confirm monozygotic twining) that we have reviewed. The case series did not provide any comparative differences in dermatoglyphic traits and kidney diseases. The Curró et al. (1982) study, regarding digital variables, showed a significantly lower incidence of radial loops and whorls in WT patients compared to normal controls. Further, they observed significantly lower TRC (both sexes) and significantly lower pattern intensity index (PII) in male patients with WT. For palmar variables, the Cummins index (Mainline index) is significantly lower in both sexes while maximal atd angle in female patients found high in contrast to controls. Gutjahr et al. (1975) showed a lower occurrence of digital arch patterns in affected cases and a slightly higher frequency of whorls in WT patients compared to controls, yet TRC remained low, as was the ab ridge count. The palmar interdigital areas III and IV showed a low occurrence of patterns compared to controls.

Gutjahr et al. (1975) further analysed dermatoglyphics in digits of the foot, and observed an increased frequency of arches, and a reduced frequency of loops and whorls in both male and female WT patients. In addition, plantar area II showed more patterns compared to controls. Both interdigital pattern III and IV found less frequency of pattern in WT patients.

### Other diseases

Our review identified several kidney diseases where dermatoglyphic features were analyzed. There are seven acro–renal–ocular syndrome cases (Halal et al. 1984), four Potter syndrome cases (Passarge 1965; Jancar 1969), four Kabuki makeup syndrome cases (Iwama et al. 1987; Philip et al. 1992), two neurofaciodigitorenal (NFDR) syndrome cases(Freire-Maia et al. 1982), a Syndactyly type V case (Robinow et al. 1982), a ring chromosome 13 syndrome case (Hoo et al. 1974), a trisomy 13 syndrome case (Pettersen 1979) and a sirenomelia case (Crawfurd et al. 1966), where different dermatoglyphic variables were described. However, these studies did not show any significant dermatoglyphic variables in patients compared to normal subjects. However, one comparative study on APCD type III patient was reported to have a lower ridge count. Unfortunately, only an abstract was available for this study. Furthermore, had the results shown that certain dermatoglyphic variables were associated with any of the above syndromic conditions, particularly those involving chromosomal aberrations (Reed and Opitz 1981), it would not have been possible to make a direct linkage exclusively between dermatoglyphics and kidney disease.

## Discussion

Our review found insufficient data to support any strong dermatoglyphic relationship with kidney diseases, in general. However, two comparative studies provided weak evidence to support an association between dermatoglyphics and WT (Gutjahr et al. 1975; Curró et al. 1982) (Table 3). Could there be some reason to suspect that this association had its origin during early foetal development? On one hand, WT is an embryonic tumor of the kidney and its exact cellular genesis is yet unclear (Pode-Shakked and Dekel 2011). It has been hypothesized that dysregulated differentiation and abnormal postnatal retention of blastemal elements in the developing kidney is the basis of oncogenesis of WT (Lovvorn et al. 2007). This observation was based on the presence of nephrogenic rests in many WT cases (Beckwith et al. 1990; Beckwith 1998; Lovvorn et al. 2007). On the other hand, dermatoglyphic development reflects the influence of environmental and hereditary factors during the first trimester (Okajima 1975; Babler 1991). Curró et al. (1982) and Gutjahr et al. (1975) evaluated dermatoglyphics in WT patients. They observed several dermatoglyphic traits in WT that differed from a comparison with non-affected people, such as low TRC (Gutjahr et al. 1975; Curró et al. 1982), low ab ridge count (Gutjahr et al. 1975) and a reduced pattern occurrence in palmer III and IV areas (Gutjahr et al. 1975). Thus, it seems possible that altered dermatoglyphic and kidney development both originated at a critical time during the embryonic/fetal period.

Curró et al. (1982) observed a very low mean TRC value in both male and female WT patients, and the mean PII is likewise very low in males compared with the values found in the control sample. Inadequate sample sizes could be one reason for the unusually high mean TRC values (male = 204.22, female = 176.29), whereas TRC in the WT patients (male = 143.5, female = 123.9) appears to be well within an expected range. Mean TRC value for populations generally is between 100 and 150 (Meier 1980). There seems to be no other explanation for this anomaly than that the Curró et al. (1982) study somehow ended up with a highly biased sample or there is the possibility that the authors actually utilized absolute ridge count rather than TRC.

Importantly, Gutjahr et al. (1975) observed a low TRC in WT patients compared with a control group in which sample size is adequate. This study did not report PII. In addition, they selected a control group from another study and did not report any demographic details. It should be pointed out that important dermatoglyphic variables such as TRC generally do differ between populations (Cummins and Midlo 1961; Meier 1980), so it is imperative that control samples be representative of the population source of the affected cases, for instance WT patients.

An important finding in Curró et al. (1982) is the higher frequency of arches among the WT males that would account, along with the lower proportion of whorls, for the lower mean values of TRC and PII in patients with WT. Further, these altered dermatoglyphic pattern frequencies might be evidence of delayed developmental timing because early ridge formation is associated with whorl patterns, late ridge formation with arch patterns and intermediate ridge formation with loop patterns, respectively (Babler 1991).

Hauser et al. (1984) compared dermatoglyphics of nine APCD type III patients with a control group and did not report any significant differences between patients and controls or patients and their healthy relatives. However, they observed that the intrafamilial comparison of the ridge counts in fingers and palms were fairly lower when plotted against their mid-parent values compared to their healthy sibs. We were able to retrieve only the abstract of this paper and it was not sufficient to comment on control group characteristics, although sample size appeared limited.

Several case reports and case series reports on dermatoglyphic variables were found for a range of kidney diseases such as Potter’s syndrome, Kabuki makeup syndrome, sirenomelia, trisomy 13 syndrome, the ring chromosome 13, acro–renal–ocular syndrome, syndactyly type V, and NFDR syndrome. Unfortunately, these cases did not provide sufficient information to conduct a comparative analysis of dermatoglyphic variables with control samples or normal populations.

A major limitation of our review is the unavailability of full text or abstract for 14 research articles. Furthermore, PubMed database did not categorise any of these studies as case control or comparative studies under “Publication Types”.

## Conclusion

According to our review, it is gratifying to learn that dermatoglyphic variables such as whorl pattern frequency and TRC have been used to investigate the uncertainty related to origin of several kidney diseases, for instance, WT and APCD type III. However, inadequate sample size and/or inconsistency between cases and control groups limits interpretation of any significant findings. Nevertheless, future studies with proper protocol, adequate cases and control groups may provide stronger evidence to diminish ambiguities related to the aetiology of kidney diseases.

## Abbreviations

CKD: chronic kidney disease
PRISMA: Preferred Reporting Items for Systematic Reviews and Meta-Analyses
MeSH: medical subject headings
APCD: adult polycystic kidney disease
WT: Wilms tumor
TRC: total ridge count
PII: pattern intensity index
